# Synergistic Effects of Sanghuang–Danshen Bioactives on Arterial Stiffness in a Randomized Clinical Trial of Healthy Smokers: An Integrative Approach to in silico Network Analysis

**DOI:** 10.3390/nu11010108

**Published:** 2019-01-07

**Authors:** Yeni Lim, Tae-Jin Song, Woochang Hwang, Ji Yeon Kim, Doheon Lee, Yong-Jae Kim, Oran Kwon

**Affiliations:** 1 Department of Nutritional Science and Food Management, Ewha Womans University, Seoul 03760, Korea; ynlim@ewha.ac.kr; 2 Department of Neurology, Ewha Womans University School of Medicine, Seoul 07985, Korea; knstar@ewha.ac.kr; 3 Department of Bio and Brain Engineering, KAIST, Daejeon 34141, Korea; wchwang.kr@gmail.com (W.H.); dhlee@kaist.ac.kr (D.L.); 4 Department of Food Science and Technology, Seoul National University of Science and Technology, Seoul 01811, Korea; jiyeonk@seoultech.ac.kr; 5 Department of Neurology, Eunpyeong St. Mary’s Hospital, The Catholic University of Korea, Seoul 06591, Korea

**Keywords:** Sanghuang mushroom, Danshen, arterial stiffness, endothelium-dependent vasodilation, randomized clinical trial, in silico network analysis, integrative approach

## Abstract

The vascular endothelium is a favorite early target of cardiovascular risk factors, including cigarette smoking. Here, we investigated the synergistic effects of Sanghuang–Danshen (SD) bioactives on vascular stiffness in a controlled clinical trial of healthy chronic smokers (*n* = 72). Relative to placebo, 4-week SD consumption at 900 mg/day improves pulse wave velocity (*p* = 0.0497), reduces systolic blood pressure (peripheral, *p* = 0.0008; brachial, *p* = 0.0046; and ankle, *p* = 0.0066), and increases endothelial nitric oxide synthase activation (*p* < 0.0001). We then mapped all differential markers obtained from the clinical data, Affymetrix microarray, and ^1^H NMR metabolomics, together with 12 SD bioactives, onto the network platform termed the context-oriented directed associations. The resulting vascular subnetwork demonstrates that ellagic acid, caffeic acid, protocatechuic acid, cryptotanshinone, tanshinone I, and tanshinone IIA are linked to NOS3, ARG2, and EDN1 for vascular dilation, implicated with arginine/proline metabolism. They are also linked to SUCLG1, CYP1A1, and succinate related to the mitochondrial metabolism and detoxification, implicated with various metabolic pathways. These results could explain the synergistic action mechanisms of SD bioactives in the regulation of vascular endothelial dilation and metabolism, confirming the potential of SD in improving vascular stiffness and blood pressure in healthy smokers.

## 1. Introduction

The vascular endothelial cells can actively and reactively participate in responding to numerous environmental stimuli and behavioral risk factors, by changing structure and function [1,2]. Typically, alterations of vascular endothelial structure and function will result in aggravating already-existent stress and promoting atherothrombotic diseases, if not correctly managed [3]. Among various risk factors for cardiovascular disease, cigarette smoking stands out as a significant behavioral health concern [4]. Cigarette smoking is mainly related to the impairment of vascular endothelium-dependent dilation, and thereby alterations in arterial stiffness and blood pressure [4,5,6,7]. Importantly, however, smoking-induced vascular endothelial change is an early and reversible phenomenon [8], providing a unique chance to evaluate the beneficial effects of lifestyle modification or drug therapies against vascular endothelium-dependent impairments.

Over the last two decades, remarkable progress has been achieved in the development of drugs that regulate vascular endothelial dysfunction and atherothrombosis, but negative results have often transpired, due to severe and unintended adverse events [9]. Alternatively, increasing attention has been paid to a range of functional foods that help to maintain or regain vascular endothelial structure and function. Multiple components in foods are expected to be working in concert with various targets in various physiological processes to modify the entire vascular system. In our previous animal and human umbilical vein endothelial cell studies, we demonstrated that a pair of herbs, including an edible mushroom “Sanghuang” (*Phellinus baumii*) and a highly valuable herb rhizome “Danshen” (*Salvia miltiorrhiza* Bunge) (i.e., Sanghuang–Danshen (SD)), contained 12 signature bioactives and ameliorated vascular endothelial dysfunction [10]. Phenolics in Sanghuang [11] and tanshinones in Danshen [12] were considered as bioactive ingredients having various biological activities. One of the mechanisms proposed in our previous study was the augmentation of endothelium-derived nitric oxide (NO) availability through the activation of endothelial NO synthase (eNOS) phosphorylation.

To advance the previous findings, in the present study, we explored the hypothesis that daily consumption of SD would be useful in improving arterial stiffness, due to the synergistic mechanisms of SD bioactives on endothelium-dependent vasodilation. To prove this hypothesis, we assessed vasomotor responses to SD consumption, by measuring blood pressure and pulse wave velocity (PWV) in a clinical trial of healthy chronic smokers. We also measured eNOS phosphorylation in erythrocytes, ^1^H nuclear magnetic resonance (NMR)-based metabolomics in urine, and microarray gene expressions in peripheral blood mononuclear cells (PBMCs). We then mapped all effects observed in the clinical trial and the analytical data of SD bioactives onto an anatomical context-specific network, termed context-oriented directed associations (CODA), which was recently developed in our previous study [13].

## 2. Subjects and Methods

### 2.1. Test Material

The SD and a color-matched placebo were kindly provided by Pulmuone Foods Co., Ltd. (Seoul, Korea). The study product was prepared, as detailed in our previous publication [10]. Briefly, the dried fruit body of Sanghuang and the root of Danshen were extracted using water and 60% ethanol in sequential order and 75% ethanol, respectively. Then, each extract was combined, filtered, concentrated, and spray-dried to yield a powder. A total of 12 bioactives were identified from Sanghuang (caffeic acid, ellagic acid, fumaric acid, hispidin, and protocatechuic acid) and Danshen (cryptotanshinone, danshensu, rosmarinic acid, salvianolic acid A, salvianolic acid B, tanshinone I, and tanshinone IIA) in our previous study, using a high-performance liquid chromatography (HPLC) equipped with a diode-array detector coupled to an ion trap mass spectrometer (Agilent Technologies, Santa Clara, CA, USA) [10]. For the standardization purpose, protocatechuic acid and tanshinone IIA were chosen as marker components at the levels of 0.45–1.5 and 50 μg/g, respectively. The study product was packed in a gelatin capsule to give a dose of 0, 600, or 900 mg SD per day.

### 2.2. Study Population

Subjects were recruited through poster advertisements. Healthy smokers (≥10 cigarettes/day), aged between 20 and 70 years with smoking history for more than 1 year, were eligible for participation. Exclusion criteria were body mass index of ≤18.5 or ≥35 kg/m^2^; a history of body weight change of ≥10% in the previous 8 weeks; a history of alcohol drinking of ≥140 g/week; the use of any medication or dietary supplements; a history of platelet dysfunction, hypertension, stroke, diabetes, thyroid disease, and any other chronic diseases likely to interfere with study participation; a history of hypersensitivity to the test material; and pregnancy or breastfeeding. The study was approved by the Institutional Review Boards of Ewha Womans University Medical Center (Seoul, Korea) and conducted according to the Declaration of Helsinki. The study was also registered in the WHO International Clinical Trials Registry Platform under the identification number KCT0001642.

### 2.3. Study Design

The study followed a randomized, double-blinded, placebo-controlled design with placebo (P), low-dose SD (L), and high-dose SD (H) groups for 4 weeks. The sample size was estimated at 24 subjects/group to detect a difference in the thrombotic tendency among groups with a statistical power of 80%, based on our previous unpublished studies with a two-sided alpha of 0.05, allowing for an attrition rate of 20%. Before entering the run-in period, a list of flavonoid-rich foods and beverages were handed out to each subject to guide restricted diets, which might be a major confounder of the assessment of arterial wall stiffness. The subjects were instructed to maintain their usual diet and lifestyle, but to refrain from eating or drinking flavonoid-rich foods and beverages. Dietary intakes and physical activities were recorded 3 days a week, including 2 weekdays and 1 weekend day, using a smartphone application. The level of compliance was monitored at the end of the study by counting the remaining capsules. At the beginning and end of the intervention period, blood pressure and arterial wall stiffness were measured; and blood and urine samples were taken after an overnight fast of ≥8 h, for western blotting, and transcriptome and metabolome analyses.

### 2.4. Measurements of Blood Pressure and Arterial Wall Stiffness

Participants rested for 15 min in a seated position, and three consecutive blood pressure readings were recorded on the non-dominant upper arm, using a noninvasive oscillometric device (Dinamap ProCare 200, GE Medical Systems, Milwaukee, WI, USA). The brachial and ankle pulse wave velocity (baPWV) was measured using a volume-plethysmographic apparatus (VP-1000 Plus system, Omron Healthcare, Tokyo, Japan). Measurements of baPWV were repeated twice, and the mean value of the right and left sides was used as the baPWV value. The radial artery pressure waveform was recorded simultaneously, using an automated tonometry system (HEM-9000AI, Omron Healthcare, Tokyo, Japan), to estimate the central arterial pressure. All measurements were determined by one trained staff, who was blinded to the study design and objectives. The average value of two measures was considered for statistical analysis.

### 2.5. Western Blotting of eNOS Phosphorylation

In erythrocytes, western blotting analysis was performed to determine eNOS and eNOS phosphorylation at Ser1177. After lying erythrocytes in a lysis buffer, the supernatant was analyzed for protein concentration by the Bradford method (Bio-Rad, Hercules, CA, USA). Equal amounts of proteins were separated by 10% sodium dodecyl sulfate–polyacrylamide gel electrophoresis and transferred to a nitrocellulose membrane (Bio-Rad). Following incubation in 5% bovine serum albumin, membranes were probed with antibodies against eNOS, phosphorylated eNOS (Ser1177) (Cell Signaling Technology, Danvers, MA, USA), and β-actin (Santa Cruz Biotechnology, Santa Cruz, CA, USA). Membrane-bound primary antibodies were visualized by horseradish peroxidase-conjugated secondary antibodies (Santa Cruz Biotechnology), and immunoreactive band intensities were quantified using a ChemiDoc XRS imager equipped with Quantity One software (Bio-Rad).

### 2.6. Statistical Analyses

All statistical analyses were performed using SAS 9.4 (SAS Institute, Cary, NC, USA). Logarithmic transformation was performed on skewed variables. Differences in the baseline characteristics were examined by one-way analysis of variance (ANOVA) or the chi-square test. The effect of the SD on each parameter was expressed as the relative change between measurements obtained at baseline and those obtained at the 4-week follow-up within each group, to consider the possible differences at baseline, among groups. Statistical comparisons were based on the one-way analysis of covariance (ANCOVA) analysis of adjusted least squares (LS) means. *p* < 0.05 was considered significant.

### 2.7. Analysis of Transcriptome and Metabolome

The PBMCs of the P and H groups were separated from blood samples using Histopaque (Sigma, St Louis, MO, USA) density gradient centrifugation. Samples were sent to the DNA Link (Seoul, Korea) for transcriptome analysis, using Affymetrix GeneChip Human Gene 2.0 ST Arrays (Affymetrix, Santa Clara, CA, USA). RNA extraction, cDNA synthesis, hybridization, and scanning of the 2.0 GeneChip were performed according to standard Affymetrix protocols. The DAT files were converted into CEL files by Affymetrix Command Console software.

The NMR spectra for urine samples of the P and H groups were acquired using a Varian Unity Inova 600 MHz spectrometer (Varian, Palo Alto, CA, USA) at Pusan National University (Pusan, Korea). Acquisition parameters were as follows: a spectral width of 24,038.5 Hz; acquisition time of 3 s, and 128 transients. Additional conditions of a relaxation delay time of 1 s and saturation power of 4 were set, to suppress massive water peaks. NMR spectra were reduced to data using Chenomx NMR Suite program ver. 4.6 (Chenomx, Inc., Edmonton, Alberta, Canada). Metabolite concentrations were annotated and quantified manually in the NMR spectra, using the Chenomx NMR Suite Professional software package ver. 4.6 (Chenomx, Inc.). The metabolomics data were imported into SIMCA-P ver. 14.1 (Umetrics, Umeå, Sweden) for multivariate analysis. Principal component analysis (PCA), orthogonal partial least squares-discriminant analysis (OPLS-DA), heat map, and metabolic pathway analysis were performed using the MetaboAnalyst 4.0 software (http://metaboanalyst.ca).

### 2.8. Integration of All Relevant Data onto the CODA Network

To construct a comprehensive component–target–pathway network, we first assembled the component–target–phenotype by searching: (1) targets of signature bioactives in SD, and (2) related phenotypes and biological markers of endothelium-dependent vasodilation and arterial stiffness. We then linked the components, targets, and phenotypes, using the shortest paths between SD bioactives and markers in the vascular subnetwork [14]. The biological marker–phenotype network is constructed by manual curation. Next, we assembled the component–target–pathway by: (1) collecting all genes and metabolites in the vascular subnetwork, (2) performing pathway enrichment analysis in a group of genes and metabolites, by the Fisher’s exact test combined with a false discovery rate of <0.01, and (3) linking the component, targets, metabolites, and enriched KEGG pathways (http://www.genome.jp/kegg/pathway.html). Finally, the component–target–pathway networks were visualized using Cytoscape 3.6.1 (http://cytoscape.org/).

## 3. Results

### 3.1. Characteristics of Study Subjects

**Figure 1:**
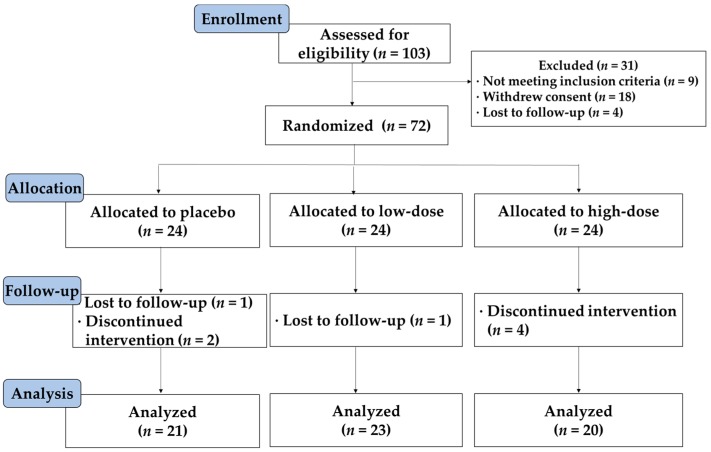
CONSORT diagram of the study. The flow of subjects from enrollment to data analysis is shown, together with significant reasons for exclusion. All subjects who completed the study were analyzed. CONSORT, Consolidated Standards of Reporting Trials.

The Consolidated Standards of Reporting Trials (CONSORT) flow diagram for this study is shown in Figure 1. Of the 72 subjects who entered the study, 64 subjects completed the study without experiencing any serious adverse events. Eight subjects were lost to follow-up (*n* = 2) or withdrew from the study due to personal reasons (*n* = 6). Data were analyzed according to both intention-to-treat and per-protocol principles. Since the results of both analyses were comparable, only the results of the per-protocol analysis of 64 samples are presented. Demographic, anthropometric, and nutritional characteristics were similar among groups, but the recommended food score value was significantly lower in the L group than in the P group (*p* = 0.0496) (Table 1). The recommended food score is a simple dietary quality score modified for a Korean diet [15].

### 3.2. Changes in Arterial Wall Stiffness, Blood Pressure, and eNOS Activation

**Figure 2:**
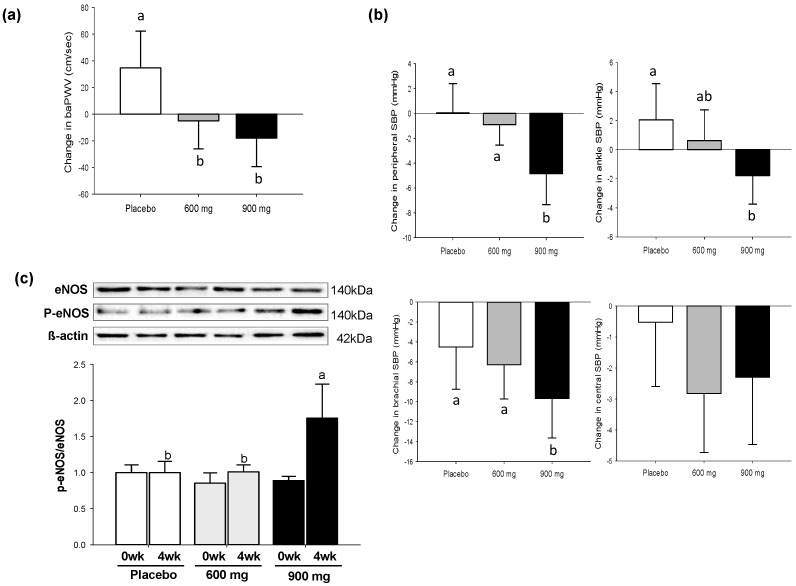
Changes in vasomotor responses to SD consumption for 4 weeks (wk): (**a**) baPWV; (**b**) SBP (peripheral, ankle, brachial, and central); (**c**) Western blotting of eNOS phosphorylation at Ser1177 in erythrocytes. SD, Sanghuang–Danshen; baPWV, brachial and ankle pulse wave velocity; SBP, systolic blood pressure; eNOS, endothelial nitric oxide synthase. Values are expressed as mean ± SE (standard error). The different letters indicate significant differences at *p* < 0.05.

As shown in Figure 2a, there was a significant treatment effect on arterial wall stiffness assessed by baPWV (*p* = 0.0497). The L and H groups showed 21% and 46% reductions in baPWV relative to the P group, respectively. Consistent results were found with the H group for peripheral systolic blood pressure (SBP) (*p* = 0.0008), brachial SBP (*p* = 0.0046), and ankle SBP (*p* = 0.0066), although the difference in the central SBP was not significant among groups (Figure 2b). Consistent with these findings, when the phosphorylated-eNOS/eNOS ratio was normalized to 1 for the P group, the H group showed a significantly higher level of eNOS phosphorylation than the P group at 4-week post-treatment (*p* < 0.0001) (Figure 2c).

### 3.3. Changes in Transcriptomics and Metabolomics

**Figure 3:**
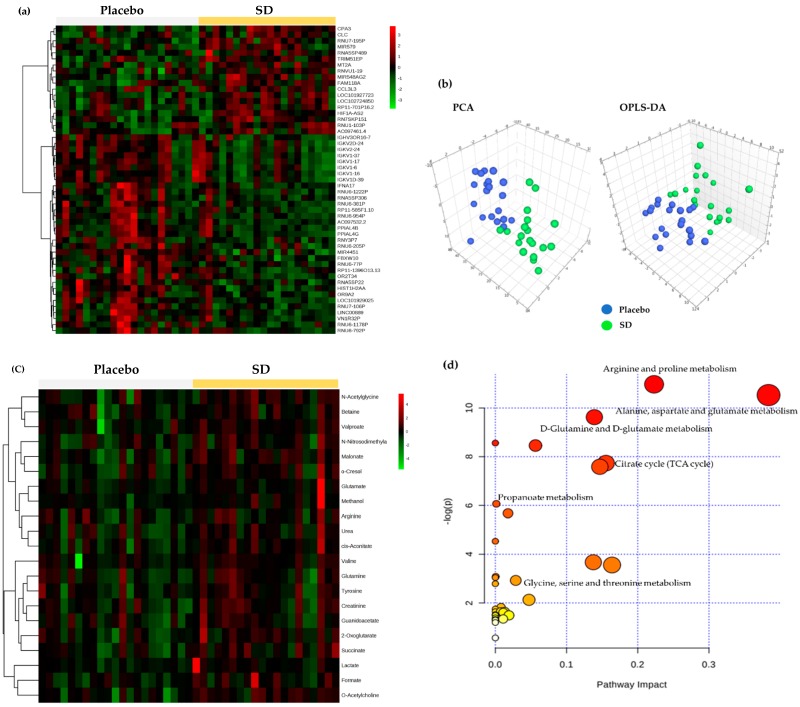
Changes in the PBMC transcriptome and urinary metabolome after placebo or high-dose SD (900 mg) consumption for 4 weeks: (**a**) heat map visualization for the PBMC transcriptome; (**b**) PCA and OPLS-DA score plots derived from urinary NMR metabolites; (**c**) heat map visualization for the urinary ^1^H NMR metabolites; (**d**) metabolic pathway analysis overview showing altered urinary ^1^H NMR metabolic pathways by SD consumption. PBMC, peripheral blood mononuclear cell; SD, Sanghuang–Danshen; PCA, principal component analysis; OPLS-DA, orthogonal partial least squares-discriminant analysis; NMR, nuclear magnetic resonance.

Microarray analysis and ^1^H NMR metabolomics analysis were carried out to show the impact of the high-dose SD consumption on gene expressions in PBMCs and urinary metabolite profiles, respectively. One hundred and sixty-nine genes met a 1.3-fold cutoff and significance threshold of *p* < 0.05, and a total of 51 genes were identified. The heat map in Figure 3a represents an overall variation of gene expressions in the two groups (placebo versus SD), indicating clear differences by SD consumption. In the meantime, PCA and OPLS-DA plots of urinary metabolomics displayed a marked separation between placebo and SD groups (Figure 3b). The OPLS-DA model in the present study was well-fitted and displayed an acceptable predictive ability (*R*^2^Y = 0.793 and *Q*^2^Y = 0.447), suggesting that SD consumption led to changes in endogenous metabolites. The following variable importance in the projection analysis identified 21 metabolites that allowed for a distinct separation between placebo and SD groups (Table S1), as visualized in the heat map (Figure 3c). Of these, the levels of 8 metabolites (*cis*-aconitate, malonate, *N*-acetylglycine, *O*-acetylcholine, succinate, urea, valproate, and 2-oxoglutarate) were statistically different between the two groups (Table S2). The results were further analyzed to perform metabolic pathway analysis using MetaboAnalyst, identifying alanine/aspartate/glutamate metabolism, arginine/proline metabolism, D-glutamine/D-glutamate metabolism, the tricarboxylic acid (TCA) cycle, propanoate metabolism, and glycine/serine/threonine metabolism as the most differential pathways (Figure 3d).

### 3.4. Synergistic Mechanisms of SD Bioactives on Vascular Endothelial Dilation

**Figure 4:**
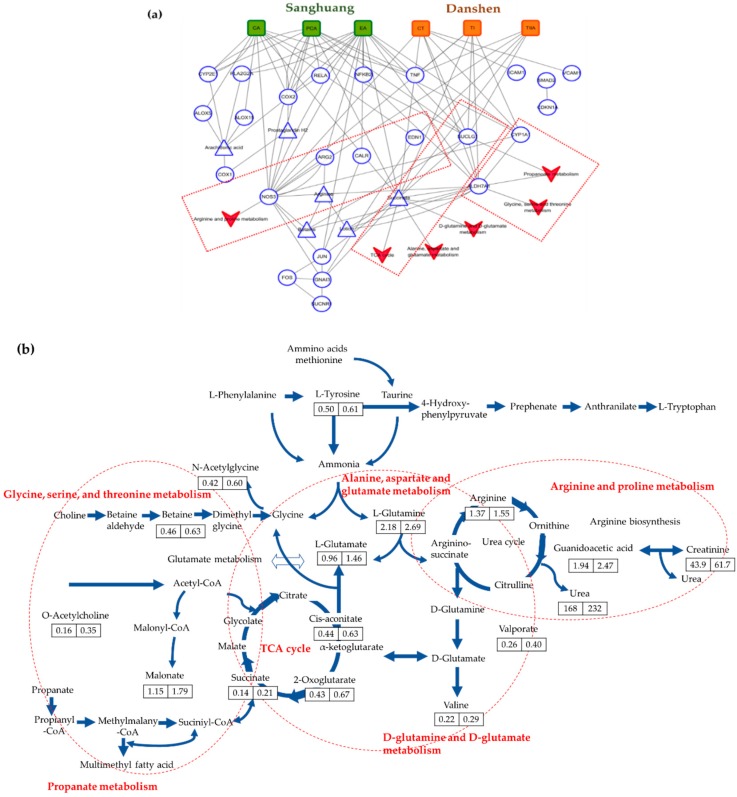
Summary of synergistic mechanisms of SD bioactives in the vascular subnetwork: (**a**) component–target protein (round) or metabolite (triangle)–pathway network (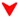); (**b**) complete sequence of six major metabolic pathways. The numbers in the box indicate the level of measured metabolites for the placebo (left) and SD (right) consumption group. SD, Sanghuang–Danshen; CA, caffeic acid; PCA, protocatechuic acid; EA, ellagic acid; CT, cryptotanshinone; TI, tanshinone I; and TIIA, tanshinone IIA. The target protein names are detailed in Table S4 (Supplementary Materials).

To facilitate the visualization and interpretation of the complex relationships between SD bioactives, target proteins, and phenotypes, we mapped the 12 signature bioactives of SD and all data obtained from the clinical trial, metabolomics, and microarray analysis on the CODA network platform. The overall network consisted of 218 nodes and 497 edges (Table S3). The further grouping by vascular endothelial dilation demonstrated that six bioactives played essential roles in this subnetwork, by interacting with the 15 targets. The target protein names are detailed in Table S4. Notably, bioactives in Sanghuang (ellagic acid, caffeic acid, and protocatechuic acid) were recognized to be highly influential components, showing the highest number of target proteins relative to those in Danshen (cryptotanshinone, tanshinone I, and tanshinone IIA). Ellagic acid had 9 targets (ARG2, CALR, COX2, CYP1A1, JUN, NO53, PLA2G2A, SUCLG1, and succinate); caffeic acid (ALOX15, ALOX5, COX2, CYP2E1, EDN1, NO53, SUCLG1, and succinate) and protocatechuic acid (ALDH7A1, COX1, COX2, CYP1A1, CYP2E1, NO53, SUCLG1, and succinate) had 8 targets. In contrast, cryptotanshinone and tanshinone I shared 5 targets (ALDH7A1, CYP1A1, NO53, SUCLG1, and succinate) and tanshinone IIA had 4 targets (EDN1, NO53, SUCLG1, and succinate). Conversely, each target had a different number of associations with bioactives in SD. The NOS3, SUCLG1, and succinate received links with all six bioactives in SD. The ALDH7A1, CYP1A1, and EDN1 also had associations with bioactives from both Sanghuang and Danshen, although fewer in number. In contrast, the ALOX5, ALOX15, ARG2, CALR, COX1, COX2, CYP2E1, and JUN received links with bioactives exclusively from Sanghuang. To understand the underlying mechanisms, we also constructed in silico metabolic pathway network corresponding to vascular endothelial dilation. The results identified that the NOS3 was connected to the arginine and proline metabolism; SUCLG1 and succinate to the TCA cycle, alanine/aspartate/glutamate metabolism, and D-glutamate/D-glutamine metabolism; and the ALDH7A1 to the glycine/serine/threonine metabolism and propanoate metabolism (Figure 4a). The complete sequence of these six major metabolic pathways is visualized in Figure 4b, showing a general increase of measured metabolites in the SD consumption group compared with the placebo group.

## 4. Discussion

In this study, we hypothesized that SD exerts beneficial effects on improving vascular wall stiffness by the synergistic mechanisms of multiple bioactives on endothelium-dependent vasodilation in the human body. In support of this hypothesis, we demonstrated a decrease in baPWV and SBP and an increase in eNOS phosphorylation in healthy chronic smokers who consumed SD for 4 weeks relative to placebo consumption. Additionally, more fundamentally, we identified that six out of twelve bioactives in SD had direct interactions with the 15 targets in the vascular endothelial subnetwork, offering insight into the synergistic actions. To date, accumulating evidence, both in vitro and in vivo, has demonstrated that a complex mixture of bioactives in plants works in concert with various molecular targets in the body, thereby exerting synergistic effects for promoting health [16]. This concept is, however, hardly employed in clinical trials, due to the inherent limitation of traditional clinical assessments. To our knowledge, we are the first group to explain how multiple bioactives in SD exert synergistic effects on vascular relaxation in human clinical trials, by adopting an integrative approach to in silico database.

While the underlying molecular mechanisms remain unclear, there are data from human studies that convincingly demonstrate the associations between chronic smoking-induced hypertension and arterial stiffness with the increased oxidative stress [17,18] and impairment of vascular endothelium-dependent dilation [4]. Of the indices that have been introduced to quantify the elastic properties of arteries, the PWV is considered as a noninvasive and simple method in the clinical practice. The PWV can be calculated based on the pulse transit time and distance traveled by a pulse between two pressure catheters placed at a known distance from one another. The aortic PWV, which measures stiffness from the carotid to the femoral artery, is the most accurate. However, it is not ideal for routine use, because some subjects may feel uncomfortable when exposing the inguinal area [19]. Therefore, in this study, we determined the baPWV, which requires placing the blood pressure cuffs on the four extremities. Sugawara et al. [19] validated that baPWV provides qualitatively similar information to aortic PWV. Moreover, Hung et al. [20] reported that baPWV is useful to predict early atherosclerotic changes in the vascular tree. Recently, an increasing number of studies have identified the promising functional foods or bioactives for vascular endothelial health using this methodology [21,22,23].

Furthermore, we also measured changes in SBP, demonstrating a statistically lowered peripheral SBP level in the high-dose SD consumption group compared with the others. However, changes in central SBP were not statistically significant. This phenomenon may be explained by the so-called SBP amplification, which means that peripheral SBP level is higher than the central SBP level, due to the reduced diameter and also the increased stiffness in the distal vessels relative to the proximal vessels [24].

To support our hypothesis of the mechanism responsible for the observed arterial stiffness and blood pressure-lowering effects of SD, we further analyzed eNOS phosphorylation in erythrocytes. NO generated from erythrocytes contributes to the intravascular NO pool, playing a prominent role in the regulation of blood flow and pathogenesis of hypertension and stroke [25]. In the present study, there was a significant increase in eNOS activation by the high-dose SD consumption relative to the placebo or low-dose SD consumption. This result is consistent with our previous findings, showing a significant increase in eNOS phosphorylation and NO production in SD-treated human umbilical vein endothelial cells [10]. Moreover, we employed an integrative approach, by merging all data obtained from this study and the 12 signature bioactives in SD with the computational network platform termed CODA, to explore the synergistic actions of SD bioactives for vascular function. The resulting network demonstrated that ellagic acid, caffeic acid, protocatechuic acid, cryptotanshinone, tanshinone I, and tanshinone IIA were the responsible components, affecting the changes in vascular function in chronic smokers. These 6 bioactives were all linked to NOS3 encoding human eNOS, suggesting that there were convergent effects of bioactives on the endothelial NO production [26], which is implicated in the arginine/proline metabolic pathway. Ellagic acid in Sanghuang was also involved in the hydrolysis of arginine to ornithine and urea by regulating ARG2, which plays a critical role in NO metabolism [27]. This finding is compatible with other independent experiments that reported the modulatory effects of protocatechuic acid [28] and ellagic acid [29] on NO availability through eNOS phosphorylation. Meanwhile, the network analysis also identified that caffeic acid in Sanghuang, and tanshinone IIA in Danshen were linked to EDN1, which is another critical target related to vascular control, as a peptide with potent vasoactive properties [30]. Collectively, our data identify SD as exerting a positive role in improving arterial stiffness and blood pressure, which is likely to be attributed to increasing NO availability through stimulation of eNOS phosphorylation. Besides, recent studies have demonstrated that proline residues play a key role as gluten epitopes recognized by the immune system in the intestine [31]. Therefore, it was suggested that the degradation of proline residues might be important in reducing gluten intolerance [32]. Given that SD is implicated in arginine/proline metabolic pathway, we advance the hypothesis that SD bioactives may play a role in alleviating symptoms of gluten intolerance.

Our network analysis also captured the stimulation of mitochondrial metabolism and detoxification as additional potential biological events related to the effects of SD against chronic smoking. The above six bioactives were directly connected with SUCLG1, the alpha subunit of succinyl-coenzyme A ligase, implicated in the TCA cycle in mitochondria, alanine/aspartate/glutamate metabolism, and D-glutamate/D-glutamine metabolism, by catalyzing the conversion of succinyl CoA to succinate and free CoA [33]. Protocatechuic acid in Sanghuang, and cryptotanshinone and tanshinone I in Danshen were recognized to bind to ALDH7A1, implicated in the glycine/serine/threonine metabolism and propanoate metabolism. ALDH7A1 is an aldehyde dehydrogenase that is thought to play a crucial role in protecting against reactive aldehydes generated during oxidative stress and the metabolism of xenobiotics [34,35]. It is also involved in protecting cells from hyperosmotic stress, by the generation of osmolytes [34,36]. Elevated oxidative stress can be caused by the coupling of hyperosmotic stress to the production of reactive oxygen species within the cell [37]. Concurrently, ellagic acid and protocatechuic acid in Sanghuang, and cryptotanshinone and tanshinone I in Danshen were linked to CYP1A1, a detoxifying cytochrome P450 enzyme that is induced by polycyclic aromatic hydrocarbons found in cigarette smoking [38].

In the present study, we also recognized that caffeic acid, ellagic acid, and protocatechuic acid in Sanghuang, but no bioactives in Danshen were connected to COX and ALOX. Both COX and LOX are the key enzymes involved in synthesizing eicosanoids, including prostaglandins, leukotrienes, and thromboxanes that are mediators of some inflammatory and allergic conditions, and platelet aggregation [39]. Eicosanoids are generated primarily through an oxidative pathway from arachidonic acid [40]. However, in the current study, arachidonic acid metabolism was not recognized as significant in the metabolic pathway analysis. This result is probably, at least partly, due to the limitation of this study of using a sole analytical platform for measuring the metabolites. Although NMR has many advantages, no single analysis technique can achieve detection of all metabolites in a biological sample. The combined use of gas chromatography–mass spectrometry might be needed for the analysis of fatty acids that are thermostable and can be derivatized to create volatile compounds [41].

## 5. Conclusions

Here, we reported for the first time that SD consumption exhibits improvement of vascular stiffness and blood pressure in chronic smokers. We also demonstrated that subsequent mapping of all data obtained in a clinical trial, Affymetrix microarray, and ^1^H NMR metabolomics, together with signature bioactives in SD, allows understanding the complex synergistic actions of SD in the field of vascular dilation, mitochondrial metabolism, and detoxification. Mainly, in this study, the integration of metabolomics into systems biology was useful to explain the underlying mechanisms. It is a limitation of this research that only NMR was used for the metabolomics analysis. Utilizing a multiplatform analysis may accelerate the study of metabolomics, providing wider information to support the synergistic effects of bioactives.

## Figures and Tables

**Table 1:** General characteristics of the participants ^1^.

Characteristic	Placebo (*n* = 21)	Low-Dose SD (*n* = 23)	High-Dose SD (*n* = 20)	*p*-Value
Gender (male/female)	19/2	21/2	19/1	0.9204
Age (years)	34.9 ± 2.5	33.0 ± 1.5	32.9 ± 2.8	0.7858
Cigarette smoking (per day)	15.6 ± 1.6	15.3 ± 1.3	14.3 ±1.1	0.7909
Alcohol consumption (yes/no)	14/7	13/10	14/6	0.6262
Total physical activity (kcal/day)	1995 ± 339	2351 ± 694	3223 ± 868	0.4281
Waist (cm)	87.8 ± 1.3	89.5 ± 2.2	84.2 ± 1.7	0.1234
Height (cm)	173.9 ± 1.4	174.1 ± 1.3	172.6 ± 1.4	0.6906
Body weight (kg)	73.4 ± 2.0	77.1 ± 2.7	69.6 ± 2.1	0.0855
Body mass index (kg/m^2^)	24.2 ± 0.5	25.4 ± 0.9	23.3 ± 0.6	0.0934
Recommended food score	22.4 ± 1.7 ^a^	16.7 ± 1.6 ^b^	20.8 ± 1.7 ^ab^	0.0496
Energy intake (kcal/day)	1492 ± 83	1421 ± 94	1488 ± 91	0.8181
Carbohydrate (g/day)	215 ± 10	195 ± 14	199 ± 13	0.5214
Protein (g/day)	51.7 ± 6.0	53.2 ± 6.7	56.0 ± 6.1	0.7676
Fat (g/day)	43.5 ± 7.5	45.7 ± 5.9	49.2 ± 6.2	0.6543
Sodium (mg/day)	2770 ± 237	2770 ± 237	2675 ± 278	0.3949

^1^ Data are shown as means ± SE (standard error). SD, Sanghuang–Danshen. The ANOVA and chi-square test were used to compare differences between groups. The different letters indicate significant differences at *p* < 0.05.

## Supplementary Materials

The following are available online at http://www.mdpi.com/2072-6643/11/1/108/s1, Table S1: List of the 21 metabolites with the VIP > 1, Table S2: Significantly altered metabolites and their related metabolic pathways, Table S3: Overall network consisting of 218 nodes and 497 edges, and Table S4: Subnetwork comprising 6 bioactives and 15 targets.
