# Supplementary material for: Synergistic Effects of Sanghuang–Danshen Bioactives on Arterial Stiffness in a Randomized Clinical Trial of Healthy Smokers: An Integrative Approach to in silico Network Analysis
Source: Nutrients. 2019 Jan 7;11(1):108. doi: 10.3390/nu11010108 (PMC6357070; doi:10.3390/nu11010108)
Supplement: Supplementary file 1 [file nutrients-11-00108-s001.zip › Table S1.docx]

Table S1. List of the 21 metabolites with the VIP > 1.

| Metabolite | VIP^1^ |
| --- | --- |
| Arginine | 1.046 |
| Betaine | 1.086 |
| cis-Aconitate | 1.363 |
| Creatinine | 1.309 |
| Formate | 1.011 |
| Glutamate | 1.057 |
| Glutamine | 1.142 |
| Guanidoacetate | 1.074 |
| Lactate | 1.089 |
| Malonate | 1.453 |
| Methanol | 1.001 |
| N-Acetylglycine | 1.285 |
| N-Nitrosodimethylamine | 1.230 |
| O-Acetylcholine | 1.556 |
| o-Cresol | 1.247 |
| Succinate | 1.454 |
| Tyrosine | 1.038 |
| Urea | 1.250 |
| Valine | 1.088 |
| Valproate | 1.259 |
| 2-Oxoglutarate | 1.329 |

^1^ VIP, variable importance plots. Variables with VIP > 1 were considered significant.
