# Supplementary material for: Synergistic Effects of Sanghuang–Danshen Bioactives on Arterial Stiffness in a Randomized Clinical Trial of Healthy Smokers: An Integrative Approach to in silico Network Analysis
Source: Nutrients. 2019 Jan 7;11(1):108. doi: 10.3390/nu11010108 (PMC6357070; doi:10.3390/nu11010108)
Supplement: Supplementary file 1 [file nutrients-11-00108-s001.zip › Table S2.docx]

Table S2. Significantly altered metabolites and their related metabolic pathways

| Metabolite | *P*-value^1^ | Related pathway |
| --- | --- | --- |
| cis-Aconitate | 0.0122 | Citrate cycle (TCA cycle); Glyoxylate and dicarboxylate metabolism |
| Malonate | 0.0483 | - |
| N-Acetylglycine | 0.0231 | - |
| O-Acetylcholine | 0.0027 | - |
| Succinate | 0.0204 | Citrate cycle (TCA cycle); Glyoxylate and dicarboxylate metabolism; Alanine, asparate and glutamate metabolism |
| Urea | 0.0040 | Arginin and proline metabolism |
| Valproate | 0.0300 | - |
| 2-Oxoglutarate | 0.0184 | D-glutamine and D-glutamate metabolism; Citrate cycle (TCA cycle); Glyoxylate and dicarboxylate metabolism; Alanine, asparate and glutamate metabolism |

^1^ *P*-values derived from the student’s *t-*test between groups, *P* < 0.05.
